# Cecal diverticulitis mimicking acute Appendicitis: a report of 4 cases

**DOI:** 10.1186/1749-7922-3-16

**Published:** 2008-04-21

**Authors:** Oguzhan Karatepe, Osman Bilgin Gulcicek, Gokhan Adas, Muharrem Battal, Yasar Ozdenkaya, Idris Kurtulus, Merih Altiok, Servet Karahan

**Affiliations:** 1 Department of General Surgery, Okmeydaný Training and Research Hospital, Istanbul, Turkey

## Abstract

Diverticulum of the cecum is a rare, benign, generally asymptomatic lesion that manifests itself only following inflammatory or hemorrhagic complications. Most patients with inflammation of a solitary diverticulum of the cecum present with abdominal pain that is indistinguishable from acute appendicitis. The optimal management of this condition is still controversial, ranging from conservative antibiotic treatment to aggressive resection. We describe four cases that presented with symptoms suggestive of appendicitis, but were found at operation to have an inflamed solitary diverticulum.

## Introduction

The cecum and ascending colon are infrequently involved in diverticulosis coli. The reported frequency is about 1 in 300 appendectomies [1,2]. The etiology of cecal diverticulitis remains unclear. Even more uncommon is a true solitary dýverticulum, which contains all layers of the bowel wall and is thought to be congenital in origin. Preoperative diagnosis is difficult because the symptoms and signs of cecal diverticulitis can mimic acute appendicitis. Diagnosis of cecal diverticulitis is difficult to make preoperatively and is thus mostly made intraoperatively. The operative treatment of cecal diverticulitis varies greatly in the literature. Here we report four cases of true solitary cecal diverticulitis managed with diverticulectomy and appendectomy, and discuss the disease in view of the literature.

### Case 1

**Figure 1:**
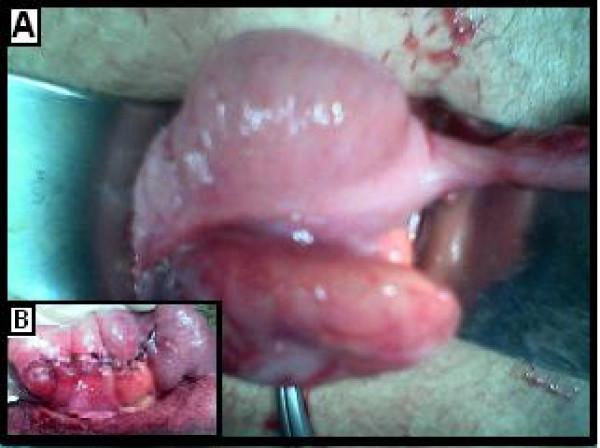
A: Intraoperative view. B: Postoperative view.

A 32-year-old-male was referred to our emergency unit with pain in the right lower quadrant of the abdomen, vomiting, and nausea that began two days prior to admission. His body temperature was 37.8°C. The physical examination showed rebound-tenderness in the lower right quadrant of the abdomen. Laboratory tests were normal except for high white blood count levels. CT scans showed a tumor in the cecum surrounded by an inflammatory plate. During the surgical exploration, an enlarged posterolateral wall was seen at the cecum, with a normal appendix. A diverticulectomy and incidental appendectomy were performed (Figure 1A,B). The pathological study established the presence of a solitary diverticulum of the cecum with acute diverticulitis.

### Case 2

**Figure 2:**
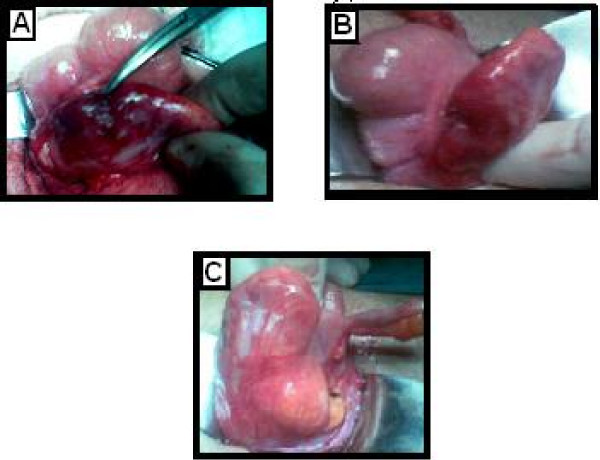
A: Case 2. B: Case 3. C: Case 4.

A 26-year-old woman was referred to our hospital after suffering from abdominal pain for four days. The progressively increasing pain started from the left hypogastric region and radiated to the lower quadrants. She had mild nausea, but no vomiting and no change in bowel habits was observed. On physical examination the bowel sounds were decreased. Upon palpation there was a muscular defense in the lower quadrants and epigastric fullness. Biochemical tests were all within the normal limits and abdominal plain X-rays revealed no abnormality. Based on the clinical findings the patient was thus considered to have acute appendicitis and underwent emergency laparotomy. At surgery, the appendix was found in the normal location with a normal appearance and a 2 × 3 cm inflamed mass was found on the anterior wall of the cecum located between the tenia libera and plica ileo-cecalis (Figure 2A). A diverticulectomy and incidental appendectomy were performed.

### Case 3

30-year-old woman presented to the Emergency Department with a pain in right lower abdomen since the day before. The pain was continuous in nature. There was no history of vomiting, fever, and no change in bowel habits. There was a history of decreased appetite and nausea. On examination, she was well oriented with a pulse rate of 90/min and BP of 120/70 mmHg. The abdomen was soft and non-distended. There was tenderness in the right iliac fossa with a positive rebound in the right iliac fossa. Bowel sounds and rectal examination were normal. Laboratory investigations revealed a Hb of 10.4 g% and total leukocyte count of 14000. Ultrasound (USG) abdomen was negative except for probe tenderness in the right iliac fossa. In view of her history and physical examination, the patient was diagnosed as having acute appendicitis and was shifted to the theatre for an appendectomy. The vermiform appendix was absolutely normal and diverticulum was found on the posterior wall of the cecum (Figure 2B). The patient underwent an appendectomy with excision of cecal diverticulum.

### Case 4

A 22-year-old woman was admitted to the hospital with right abdominal pain, nausea and vomiting. She mentioned that the abdominal pain had migrated to the right lower abdomen 12 hours after onset of periumbilical pain. On physical examination, she was subfebrile (37.5°C) and had right lower abdominal tenderness with physical signs of peritonitis. Bowel sounds and rectal examination were normal. The white blood count was 14200/mm3. Plain abdominal x-ray and USG findings were normal. A preoperative diagnosis of acute appendicitis was made and a laparotomy was performed through a right lower quadrant transverse incision. The patient was taken to the theatre where a standard open appendectomy was started. During the operation a suspicious mass was found in the lower ascending colon (Figure 2C). The appendix was visualized and found to be normal. A diverticulectomy was performed.

## Discussion

Diverticulosis is extremly common in the United States and Europe. It is estimated that half of the population older than age 50 years has colonic diverticula. The true prevalence

of colonic diverticulosis is difficult to ascertain, however it appears that about 8.5% of people in western countries are afflicted [1]. The sigmoid colon is the most common site of diverticulosis. Right-sided diverticula occur more often in younger patients than do left-sided diverticula, and are more common in people of Asian descent than in the other populations. The majority of colonic diverticula are false diverticula in which the mucosa and muscularis mucosa have herniated through the colonic wall [2]. Solitary diverticulum of the cecum is thought to be a congenital lesion arising as a sacular projection during the sixth week of embryonic development [2,3].

Most patients with right side diverticula are asymtomatic. However, diverticulitis does occur occasionally. Because patients are young and present with right lower quadrant pain, they are often thought to suffer from acute appendicitis, and the diagnosis of right-sided diverticulitis is subsequently made in the operating room. It is difficult to differentiate cecal diverticulitis from acute appendicitis. More than 70% of patients with cecal diverticulitis were operated on with a preoperative diagnosis of acute appendicitis [3]. The correct preoperative diagnosis was made in only 5.3% of 318 patients, according to the report of Wagner and Zollinger [4]. A number of reviews report that the incidence of a correct intraoperative diagnosis oscillates between 65 and 85% [4]. In all of our cases except one, the preoperative diagnosis was acute appendicitis.

Ultrasound and computer tomography (CT) have both been evaluated in the diagnosis of right-sided diverticulitis. Chou et al [5] reviewed 934 patients with clinically indeterminate right-sided abdominal pain who went on to have an abdominal ultrasound. They reported that ultrasound has demonstrated a sensitivity of 91.3%, a specificity of 99.8% and overall accuracy of 99.5% in the diagnosis of cecal diverticulitis [5]. CT scans have a sensitivity and specificity of 98% in the diagnosis of acute appendicitis, and are highly cost-effective; hence, some authors suggest its routine use for abdominal pain in the right lower quadrant, which would probably reduce surgeries and hospital stays [5,6]. Recognition of specific imaging findings enables the radiologist to make the correct diagnosis and helps in establishing the appropriate surgical or medical therapy, thus avoiding unnecessary exploration or surgery for some of these nonsurgical conditions mimicking acute appendicitis. If preoperative examination suggests cecum diverticulitis, the most important diagnostic tool is the CT. The CT findings were similar to those of left side diverticulitis, including focal pericolonic inflammation, diverticula, colonic wall thickening, thickening of the adjacent fascia, and extraluminal mass effect.

In patients with preoperative diagnosis of cecal diverticulitis without signs of peritonitis, medical treatment with antibiotics may be sufficient [6,7]. In our cases, almost all of our patients had no history of appendectomy, so appendicitis was the main clinical suspicion in these cases, which led to the operative exploration of the abdomen. An intraoperative diagnosis is difficult upon initial exploration. In addition, when the diagnosis is made intraoperatively, the surgical management of the disease is controversial. Conservative management with antibiotics has been suggested for cecal diverticulitis diagnosed intraoperatively, but most surgeons recommend resection [8,9]. In the presence of an inflammatory mass, diverticulectomy is usually impossible, and colectomy is required. A literature review of 279 cases of surgically treated cecal diverticulitis found no mortality after ileocecal resection, but a mortality rate of 1.8% after right hemicolectomy [10,11]. Fang et al. recommend wide resection, since 29% of patients undergoing only appendectomy in their study had recurrent episodes of right diverticulitis, with 12.5% of them requiring a later right hemicolectomy [11]. In all of our patients, a diverticulectomy and incidental appendectomy were performed and postoperative periods were uneventful.

In conclusion, Preoperative diagnosis of cecal diverticulitis is important in order to decide how to manage to this condition. During the surgical procedure, if the diagnosis of acute appendicitis is in doubt, further exploration should be performed. We recommend diverticulectomy as a safe and adequate treatment for cecal diverticulitis. However, if the histopathological examination of the specimen reveals the presence of colonic cancer, a right hemicolectomy can always be performed.
